# Engaging in extreme activism in support of others’ political struggles: The role of politically motivated fusion with out-groups

**DOI:** 10.1371/journal.pone.0190639

**Published:** 2018-01-05

**Authors:** Jonas R. Kunst, Beverly Boos, Sasha Y. Kimel, Milan Obaidi, Maor Shani, Lotte Thomsen

**Affiliations:** 1 Department of Psychology, University of Oslo, Oslo, Norway; 2 Center for Research on Extremism, University of Oslo, Oslo, Norway; 3 Department of Political Science, Aarhus University, Aarhus, Denmark; 4 Department of Psychology, Harvard University, Cambridge, Massachusetts, United States of America; 5 Department of Psychology, Uppsala University, Uppsala, Sweden; 6 Jacobs University Bremen, Bremen, Germany; University of Melbourne, AUSTRALIA

## Abstract

Humans are a coalitional, parochial species. Yet, extreme actions of solidarity are sometimes taken for distant or unrelated groups. What motivates people to become solidary with groups to which they do not belong originally? Here, we demonstrate that such distant solidarity can occur when the perceived treatment of an out-group clashes with one’s political beliefs (e.g., for Leftists, oppressive occupation of the out-group) and that it is driven by fusion (or a feeling of oneness) with distant others with whom one does not share any common social category such as nationality, ethnicity or religion. In Study 1, being politically Leftist predicted European-Americans’ willingness to engage in extreme protest on behalf of Palestinians, which was mediated by fusion with the out-group. Next, in Study 2, we examined whether this pattern was moderated by out-group type. Here, Norwegian Leftists fused more with Palestinians (i.e., a group that, in the Norwegian context, is perceived to be occupied in an asymmetrical conflict) rather than Kurds (i.e., a group for which this perception is less salient). In Study 3, we experimentally tested the underlying mechanism by framing the Kurdish conflict in terms of an asymmetrical occupation (vs. symmetrical war or control conditions) and found that this increased Leftist European-Americans’ fusion with Kurds. Finally, in Study 4, we used a unique sample of non-Kurdish aspiring foreign fighters who were in the process of joining the Kurdish militia YPG. Here, fusion with the out-group predicted a greater likelihood to join and support the Kurdish forces in their fight against ISIS, insofar as respondents experienced that their political orientation morally compelled them to do so (Study 4). Together, our findings suggest that politically motivated fusion with out-groups underpins the extreme solidary action people may take on behalf of distant out-groups. Implications for future theory and research are discussed.

## Background

*“I have left my family and a comfortable life in Britain to risk suffering the most horrific death at the hands of IS*. *I’ve got a grenade in my pocket and I’ll blow myself up and take them with me*.*"-* Macer Gifford, White British citizen who joined the Kurds’ fight against ISIS [1]

*“When I come back from Palestine*, *I probably will have nightmares and constantly feel guilty for not being here*, *but I can channel that into more work*. *Coming here is one of the better things I’ve ever done*.*—*Rachel Corrie, a White American activist who was killed in the Gaza Strip while trying to block an Israeli armored bulldozer [2]

It has been estimated that up to 35 thousand individuals from across Europe and the United States volunteered as soldiers in the Spanish Civil War against dictator Francisco Franco [3, 4]. What these foreign fighters had in common was a willingness to risk their lives for an ethnic and national group that they did not belong to, joining them in a lethal armed conflict in which they were not originally involved. As presented in the above quotes, such extreme and risky support for the political struggles of out-groups can also be observed today. For instance, it has been estimated that several hundred foreign fighters have voluntarily joined the Kurdish YPG forces (“People’s Defense Units”) in their fight against the Islamic State of Iraq and Syria (ISIS) [5]. Similarly, numerous Western activists, such as Rachel Corrie, have risked their lives or even died supporting the rights of Palestinians. What drives such people to engage in this type of extreme solidary behavior to support out-groups in political conflicts?

A host of studies has shown that a feeling of “oneness” with the in-group predicts extreme behavior on their behalf [6–9]. While previous research has found that this phenomenon can occur even with larger, abstract groups to which one belongs (e.g., one’s religious or national group) [7, 10], it is an open question if the psychological role of fusion processes is so fundamental and flexible that solidary actions on behalf of groups one does not initially belong to will also be motivated by it (but see [11] for a potential exception within the gender domain). That is, when people come to engage in extreme solidarity on behalf of out-groups of which they are not a priori members, is this also driven by perceptions that they are “one” with them and, if so, what are the conditions under which this occurs? Addressing this question is also addressing the possibility of social change: It is blatantly the case across cultural history that sometimes people and groups do join new alliances on behalf of which they may risk their lives, even if nationhood, ethnicity and religion are categories to which people are especially deeply fused today. Building on previous work on the relationship between extreme group behavior, identity fusion, and sacred values [12–15], here we predict that people are especially likely to fuse with a distant group if the way it is treated clashes with one’s fundamental ideological and political beliefs [12].

### The potential role of fusion with out-groups in extreme solidary action

Distinct from normative social action (e.g., joining peaceful protests), extreme or non-normative social action often involves confrontations with militarized police or armed forces. Generally, people are more willing to engage in this kind of risky, self-sacrificing, and potentially life-threatening behavior for people with whom they share close relational ties [6, 16] or a common dysphoric and negative past [17, 18]. According to the theory of identity fusion [7], such extreme group behavior occurs because people experience being ‘one’ or fused with a group and, thus, perceive an overlap between their personal selves and their group. Support for this prediction has been observed on various measures of extreme support for one’s own group, including a willingness to fight and die for others, self-sacrifice in the trolley dilemma, and joining of revolutionary battalions and resistance movements [7, 9, 10, 16, 19–23].

Although similar to social identity, identity fusion is conceptualized as a distinct construct. As Hogg et al. [24] put it, in terms of social identity “the basic idea is that a social category (e.g., nationality, political affiliation, sports team) into which one falls, and to which one feels one belongs, provides a definition of who one is” (p. 259). Hence, different to personal identities, social identity refers to the part of the self-concept that is defined by membership in social groups [25]. In contrast to social identity, identity fusion denotes a state where individuals experience a visceral feeling of overlap between their personal and social selves [7]. In other words, they become *one* with the group. Moreover, although various subcomponents of social identity are conceptually similar to identity fusion at first glance (e.g., in-group homogeneity, solidarity), these are also conceptualized in distinct ways from what is the case in formulations of identity fusion. For instance, while the solidarity dimension of social identity involves feeling “a bond with,” “solidarity with” or “committed to” the in-group [26], one’s personal self and social identity are thought to still remain independent. By contrast, identity fusion involves feeling “immersed” or “one” with the group [20], such that the boundaries between both identities become permeable, allowing them to fuse to one [7].

Previous work on identity fusion highlights that people are likely to fuse with a group especially when they perceive other group members as kin [6, 27]. Hence, unsurprisingly, identity fusion is common for smaller groups in which group members in fact have strong face-to-face or even genetic relational ties (e.g., siblings, friends [28]). Yet, research suggests that people also tend to project such kinship-like processes to larger groups [9, 27, 29], for instance ethnic groups with which one may perceive a shared essence or bloodline. Arguably, as a result of these “fictive kin” perceptions [29], identify fusion can also be observed within more abstract higher-order groups, or *imagined communities* [30], for which actual genetic relatedness or even personal relations between most members is low such as cultural and national groups–processes that may generalize even to political and gender groups [8, 11, 31, 32]. Indeed, it has been suggested that people may show such “extended fusion” based on various abstractions such as a common political cause or ideology, in some sense treating people as if they were kin even when they have had no, or little, actual connection or face-to-face contact with other group members [7].

In short, a key insight of fusion theory is that the communality felt with larger, abstract groups is not simply the result of arbitrary social construction and communication, but shaped by the evolved logic of altruism directed towards kin ([33]; see also [34] for a similar analysis). This proclivity to recruit and apply kinship-like fusion processes, even at high levels of abstraction with groups that are not defined in terms of any shared biological essence, point to the fundamental importance of fusion-processes in navigating intergroup relations and conflicts. However, in previous demonstrations of extended fusion, participants’ ethnicity was often nested within, overlapped or substantially covaried with the abstract higher-order group in question (e.g., native Spaniards fusing with Spain; Israeli Jews fusing with Judaism; ethnic Poles fusing with the Polish national group or their religion in a country where the vast majority of Catholics are native Poles; [16, 21, 22, 32, 35]). Because ethnicity, religion and nationality often co-occur and are conceptually intertwined [36–38], this suggests that some perceptions of shared ancestry or biological essence might already be present in cases of extended fusion with national or religious groups for instance, or at least that the ambiguity of such groups lends itself readily for the extension of kinship-like processes driven by perceptions of shared essence and ancestry. Hence, a stronger test of the fundamental role of psychological fusion processes for extended solidarity would be if they also account for solidary extreme action for out-groups that appear to share *no* factual kinship, bloodline or biological essence with oneself.

Of course, in some sense the idea of fusing with an out-group is paradoxical: If you become one and part with a group, surely it must now be your in-group? This line of argument refers to the outcome of fusion processes, but it does not address if and why people come to fuse with new groups that they are not initially part of in the first place (in contrast to their own national, ethnic and often religious groups). Because ethnic, religious and national groups may be perceived to share biological ancestry, here we test if fusion processes also underpin solidarity with ethnic out-groups. If this is the case, it would suggest that kin-like perceptions of oneness underpin solidarity even with groups that one does clearly not share any kin-like common biological ancestry or essence with. This in turn would suggest that fusion processes of perceived oneness, although possibly ultimately rooted in biological kin altruism, flexibly underpin the formation and joining of new groups that occur in social change.

Hence, while individuals likely come to perceive the out-group as their in-group *after* fusing with it, when we focus on fusion with ethnic out-groups in the present studies, we refer to the process that makes it possible to fuse with a group that initially constituted–and from an outsider’s perspective still may be considered as–an out-group. An average person who is White American or Norwegian has no Palestinian or Kurdish ancestors and, thus, is likely to perceive neither as their racial/ethnic in-group. Yet, as we aim to demonstrate, Norwegians and White Americans may come to feel fused with Palestinians or Kurds. The present research investigates a process that motivates such fusion with out-groups across ethnic boundaries. To ensure that we indeed assess fusion across ethnic groups, we ask participants to indicate their ethnic group membership in each study. Moreover, to rule out the possibility that fusion with the out-group is simply due to perceptions of shared values with the out-group, we test and control for such a potential confound in one study.

### The present research

In four studies, we investigated antecedents of fusing with outgroups and its impact on extreme solidary support. Based on recent research suggesting that individuals’ fusion with groups may be motivated by a desire to protect sacred values [12, 14], we predicted that people would fuse especially strongly with out-groups whose treatment is perceived to clash ideologically with one’s own core political views. Hence, because the oppression of groups in asymmetric conflicts via occupying powers stands in sharp disagreement with Leftist political ideology [39–41], Leftists would be expected to fuse with oppressed out-groups, motivating them to act on their behalf. While the term “Leftist” is a broad construct, the general left-right continuum is the primary way to describe and categorize political beliefs in many, if not most, parts of the world [42, 43]. Yet, despite this universality, the specific ideological content associated with this dimension varies between contexts [39, 44]. However, at least in liberal societies such as those of Western Europe and North America, from which most of our participants were recruited, being Leftist typically involves a concern for harm being done against others and equality for all [39, 45, 46]. Against this background, we tested the specific prediction that Leftists should fuse in particular to out-groups that are perceived to be violently oppressed, using a variety of populations and out-groups, and correlational and experimental designs. First, using a sample of European-Americans, we tested whether having a Leftist political orientation predicts more fusion with Palestinians and subsequently more support for non-normative extreme protests on their behalf. Next, in a sample of Norwegians, we tested whether Leftists are more fused with out-groups who are perceived to be maltreated in a way that ideologically clashes with central Leftist values (i.e., the Palestinians living under oppressive occupation) compared to out-groups whose treatment tends to not be seen as clashing with these values to the same degree (i.e., the Kurds). In a third study, we aimed to causally test whether framing the treatment of an out-group as a violation of Leftist political ideology would increase the extent to which this political orientation predicts fusion with the out-group. Specifically, we tested whether experimentally framing Kurds as living under oppressive occupation, rather than being involved in a symmetric war, might make Leftists European-Americans fuse with Kurds more and, thereby, increases their willingness to engage in non-normative extreme protest on their behalf. We included measures of normative as well as non-normative extreme protest in the first three studies to allow us to test whether the effects of fusion with out-groups are especially strong on non-normative extreme actions in line with previous research reviewed above. Finally, using a unique sample of aspiring foreign fighters in the very process of joining the Kurds in their fight against ISIS, we tested whether having a Leftist political orientation creates more fusion with the Kurdish out-group and, thus, more willingness to fight and die for this group.

## Study 1

In the U.S., political engagement on behalf of Palestinians has become an important part of the Leftist political agenda. Indeed, former 2016 presidential candidate Bernie Sanders was a vocal supporter of Palestinian rights. Moreover, according to a recent survey, 29% of self-identified Liberal Democrats sympathize more with the Palestinians than with the Israelis, compared to only 7% among self-identified Republicans [47]. This sympathy among the Left can be observed in form of political movements and protests against Israel and the Israeli occupation including “Boycott, Divestment and Sanctions (BDS).” Here, we tested whether a Leftist political orientation among European-Americans is associated with higher perceived fusion or “oneness” with Palestinians, and whether this fusion, in turn, predicts extreme activism on this group’s behalf.

To estimate the unique effects and relative strength of this political orientation–fusion with the out-group pathway, we also controlled for a selection of alternative predictors of the social identity [48] and social dominance models of collective action [49, 50]. Specifically, meta-analytical evidence has validated three major social identity predictors of social action [48]: 1) politicized identities such as activist identities, 2) affective reactions to injustice such as anger, and 3) political efficacy or the belief that one’s efforts are likely to produce social change. While these factors have mostly been studied in terms of collective action supporting one’s own group, some recent research has shown that efficacy beliefs and moral outrage (i.e., anger) can also predict solidary action for disadvantaged out-groups (e.g., [51]). Hence, in this study, we controlled for politicized social identity and its potential pathways through anger and perceived efficacy. In previous identity fusion research, social identification has often been measured with items such as “When someone praises my country, it feels like a personal compliment” or “When someone criticizes my country, it feels like a personal insult” [20, 23]. As these items may be seen as measuring outcomes of social identification rather than social identification per se, in our research, we used a more direct social identification measure developed by Hornsey et al. [52]. Importantly, this scale measures a politicized activist identity which, according to previous research is more predictive of collective action than are non-politicized identities [48]. Hence, it constitutes an appropriate control variable in the context of research on solidary collective action for out-groups. Importantly, here we use the term “solidary” to explicate that we focus on collective action in support of out-groups (rather than one’s in-group), and not to refer to the emerging literature seeing solidarity as a sub-component of social identity [26].

Finally, it has recently been argued that people can engage in solidary action with the motivation to attenuate or strengthen between-group hegemony [50], as captured by their Social Dominance Orientation (SDO) [53]. For instance, Stewart et al. [50] found that the higher non-Arab foreigners’ SDO levels were, the lower their support was for the Arab Spring protesters. This effect was mediated by a perception that Arabs’ lack the competence to rule their own countries (but see [54]). Hence, we also controlled for SDO and a measure of perceived Palestinian competence.

### Materials and methods

#### Participants

A total of 201 European-Americans, who were not Jewish and who were living in the U.S., participated in a study on “political conflicts” through Amazon MTurk (*M*_*age*_ = 34.6, *SD*_*age*_ = 10.00; men: 56.7%). The focus on non-Jewish European Americans was important for two reasons: First, DNA ancestry research suggests that Jews and Palestinians are genetically related and that knowledge about this may positively impact intergroup attitudes [55]. Hence, Jewish participants may have perceived an overlap between their own racial/ethnic group and the Palestinian out-group due to shared perceived kinship. Second, although American Jews are not directly involved in the Israeli-Palestinian conflict, they often show emotional attachment to the Israeli state, which is a majority Jewish nation. Because our focus was on fusion with a distant ethnic out-group to which one does not belong to nor is involved in a conflict with, we focused on non-Jewish European-American participants here. Participants were paid $1 for participation. This and all remaining studies were approved by the Institutional Review Board of the Departments of Psychology at the University of Oslo (Nr. 1790201). Unless stated otherwise, they completed the following measures on 7-point Likert-type scales, ranging from 1 (*totally disagree*) to 7 (*totally agree*):

#### Leftist political orientation

In all studies presented in this paper, we assessed political orientation using a single-item indicator. This approach is common in social-scientific survey research (e.g., European Social Survey, World Value Survey, General Social Survey), arguably because it captures political orientation parsimoniously and equivalently across various social and demographic groups [43] and has high predictive validity [56]. Specifically, we used the validated 10-point format [57] to assess political orientation in all studies. For each study, we used the terms most common in the respective political context to denote the endpoints of the scale. That is, the endpoints were “very liberal”—“very conservative” in the US (Studies 1 and 3) and “extremely left-wing”—“extremely right-wing” in the Norwegian student population (Studies 3), while we use a combination of both terms (i.e., “very liberal/left-wing”—“very conservative/right-wing”) in the last international study (Study 4). Hence, in the present study, which was conducted in the US, participants rated their political orientation on a scale ranging from 1 (*very liberal*) to 10 (*very conservative*). This scale was reverse-scored so that higher values indicated more Leftist political orientation.

#### Social dominance orientation

The new SDO_7_ scale [58] was used to measure social dominance with 16 items (α = .96) such as “It’s probably a good thing that certain groups are at the top and other groups are at the bottom”.

#### Activist identity

We adapted a four-item scale developed by Hornsey et al. [52] to measure Palestine-activist identity (e.g., “I identify as a Palestine-activist”; α = .94).

#### Fusion with the out-group

Participants completed the seven-item identity fusion scale developed by Gómez et al. [20]. To measure fusion with the out-group, the scale was adapted such that all items were framed towards the Palestinian out-group (i.e., “I am one with the Palestinian people”; α = .94). As in Gómez et al. [20], factor analysis showed that the identity fusion and social identity measures represented distinct constructs.

#### Palestinian competence

Participants completed the item, “The Palestinian people are competent enough to govern themselves”, adopted from Stewart et al. [50] on a 10-point scale ranging from 1 (*totally disagree*) to 10 (*totally agree*).

#### Political efficacy

As in Saab et al. [51], we used four items to measure participants’ efficacy beliefs (α = .96). For instance, participants indicated whether they believed that protesting would result in “achieving justice in Palestine” on a 7-point scale ranging from 1 (*not at all*) to 7 (*extremely*).

#### Anger

Three items from Ufkes et al. [59] were used to measure the degree to which participants felt anger, frustration and irritation regarding how Palestinians are treated (α = .98).

#### Normative protest intentions

As in Saab et al. [51], we asked participants how many of the next ten organized protests for justice in Palestine they were likely to take part in. Response options ranged from 0 to 10.

#### Willingness to engage in non-normative extreme protest

Willingness to engage in non-normative extreme protest (abbreviated as ‘extreme protest’ in the analyses) was measured with two items adopted from Simon et al. [60, 61] and slightly adapted to the present context: “I would participate in Palestine protests involving confrontations with the police” (original item: “I would participate even in a protest action which may involve a confrontation with the police”) and, “Sometimes violent protest for Palestine is the only means to wake up the public” (original item: “Sometimes violent protest is the only means to wake up the public”), *r*(199) = .50, *p* < .001.

### Results

As displayed in Table 1, fusion with the out-group and activist identity were related to higher normative protest intentions and willingness to engage in extreme protest. Surprisingly, SDO was unrelated to both solidary action measures when considered as a full scale (.155 < *ps* < .583) or as separate dominance and anti-egalitarianism subscales (.112 < *ps* < .894). SDO and the potential mediator of perceived competence were therefore not included in the following models.

**Table 1:** Means, Standard Deviations and Correlations between variables in Study 1 are displayed.

	*M* (*SD*)	2.		3.	4.		5.		6.		7.		8.		9.	
1. Fusion with Out-group	1.83 (1.09)	.61	***	.01	.17	*	.20	**	.31	**	.11		.36	***	.50	***
2. Activist Identity	1.75 (1.17)			-.01	.15	*	.30	***	.37	***	.10		.49	***	.52	***
3. SDO	2.54 (1.43)				-.51	***	-.43	***	-.21	**	-.29	***	.10		-.04	
4. Leftist Political Orientation	6.63 (2.51)						.36	***	.24	***	.23	**	.06		.15	*
5. Anger	4.24 (1.76)								.41	***	.37	***	.16	*	.31	***
6. Efficacy	2.99 (1.53)										.23	**	.23	**	.24	**
7. Palestinian Competence	7.04 (2.38)												.06		.15	*
8. Normative Protest	1.71 (1.96)														.56	***
9. Extreme Protest	1.77 (1.17)															

Note

*p < .05.

**p < .01.

***p < .001.

**Fig 1:**
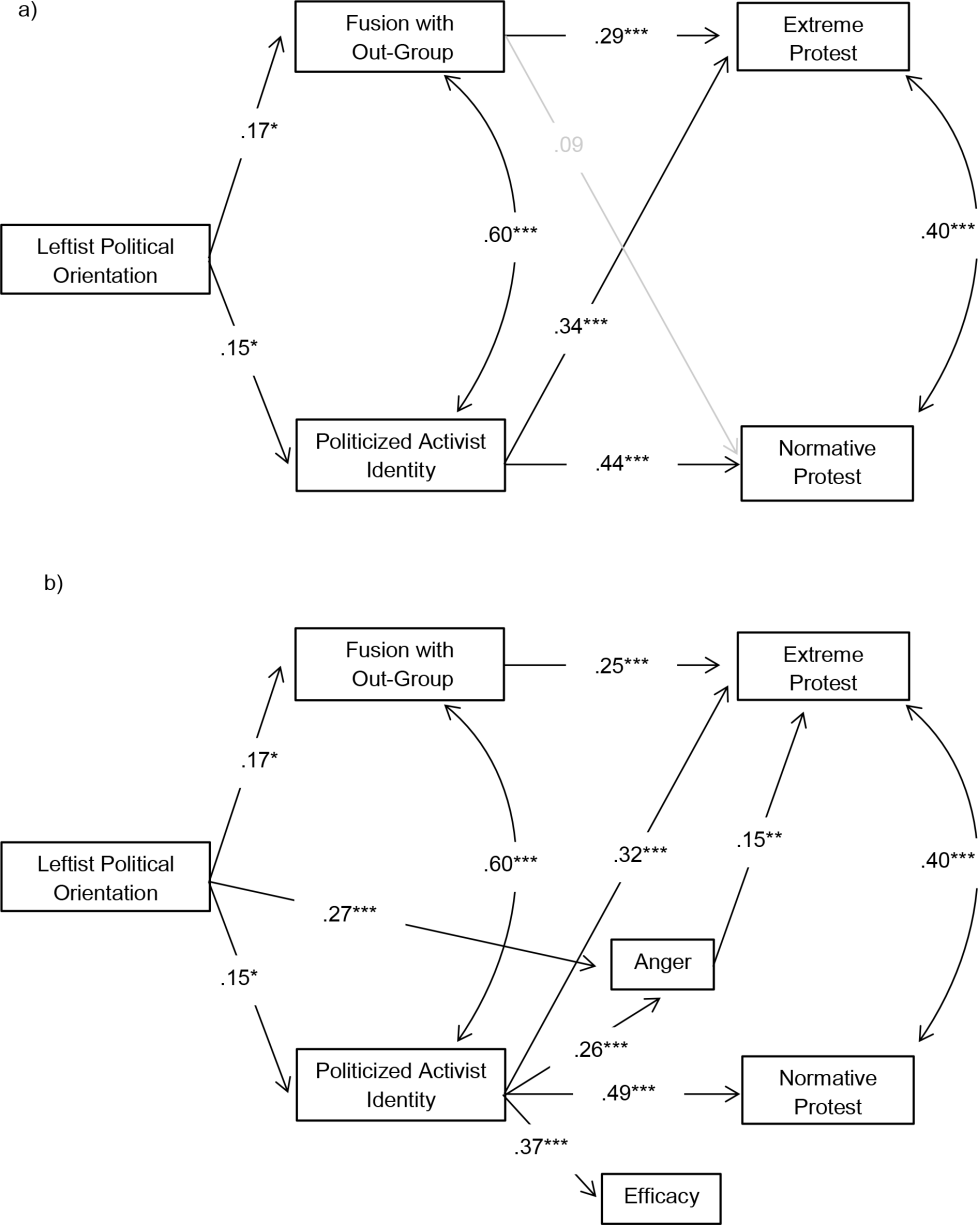
Simple (a), and more complex and fitted (b), mediation models for Study 1 are displayed. Non-significant paths are displayed in grey. The correlation between anger and efficacy in Model b) is not displayed for purposes of presentation: *r*_anger, efficacy_ = .30, *p* < .001.

In the first stage, we tested a simple model where Leftist political orientation predicts fusion with the out-group and activist identity, which both predict extreme and normative protest in turn (see Fig 1A). Here and in all other path models tested in this paper, maximum likelihood estimation was used to account for potential non-normality of the dependent variables [62]. In the well-fitting model, χ^2^ (*df* = 2, *N* = 201) = 1.38, *p* = .502, *Root Mean Square of Approximation (RMSEA)* < .001, *Comparative Fit Index (CFI)* = 1.00, *Root Mean Square Residual (RMR)* = .05, Leftist political orientation positively predicted fusion with the out-group and activist identity. Fusion with the out-group, in turn, positively predicted extreme protest, while activist identity predicted both extreme and normative protest. Bootstrapping with 5,000 random resamples showed that leftist political orientation indirectly predicted more extreme protest mediated by fusion with the out-group (β = .05, 95% CI [.01, .11], *p* = .006) as well as activist identity (β = .05, 95% CI [.01, .13], *p* = .015). These effects did not differ in strength (*p* = .848). Moreover, Leftist political orientation had an indirect positive effect on normative protest intentions that was mediated only by activist identity (β = .07, 95% CI [.01, .17], *p* = .021).

We further tested a reversed model in which activist identity and identity fusion had effects on political orientation which, in turn, was expected to predict extreme and normative protest. However, in this fully-saturated model, political orientation had no effect on either dependent variable and, hence, did not mediate the effects (see S1 Fig in the supplementary information).

We also estimated a more complex model in which the effects of activist identity on normative and extreme protest were hypothesized to be further mediated by anger and political efficacy, following the social identity approach of collective action. However, in the poorly-fitting model, χ^2^ (*df* = 7, *N* = 201) = 29.15, *p* < .001, *RMSEA* = .126, *CFI* = .938, *RMR* = .297, only anger mediated the effects of activist identity on extreme protest, while efficacy did not mediate at all. When we fitted the model by deleting the non-significant paths and adding a direct path from leftist political orientation to anger, model fit was satisfactory, χ^2^ (*df* = 6, *N* = 201) = 13.54, *p* = .140, *RMSEA* = .050, *CFI* = .987, *RMR* = .015 (see Fig 1B). In this fitted model, the effect of Leftist political orientation on extreme protest that was mediated by activist identity was further mediated by anger, resulting in a weak indirect relationship (β = .01, 95% CI [.001, .02], *p* = .012). Moreover, political orientation had an indirect positive effect on extreme protest that was mediated by anger (β = .04, 95% CI [.02, .08], *p* < .001).

### Preliminary discussion

This first study provided initial support that fusion with out-groups mediates the effect of political orientation on extreme solidary action on behalf of out-groups in need. Specifically, the more politically Leftist people were, the more they fused with the Palestinian out-group which, in turn, resulted in higher willingness to engage in non-normative extreme protests. This relationship remained stable even when controlling for alternative variables known for predicting solidary action. Why then do people fuse and show extreme support for some out-groups in political conflict but not for others? Next, we aimed to address this question while also confirming the robustness of our proposed political orientation–fusion with outgroups pathway by replicating our initial findings in a different cultural context.

## Study 2

Despite its far geographical distance from the Middle East, Norway has a long history of political engagement on behalf of Palestine. For instance, Norway played a central role in the 1993 Oslo peace-treaty and provides more foreign aid to Gaza and the West Bank relative to its population size or GDP than any other country does [63]. At the grassroots level, this Palestine support can be observed in form of Leftist political protests against the way Palestinians are treated, sometimes resulting in violence and vandalism (see [64, 65]). In contrast, a group that finds itself in a similar situation as the Palestinian people but receives far less support from Norway are the Kurds. In their modern history, the Kurdish minority living in Turkey, Iraq, Iran and Syria have been frequent victims of violent oppression [66]. Some of the most extreme examples include Saddam Hussain’s 1988 genocide in the Kurdish city of Halabja, in which chemical weapons killed thousands of Kurdish civilians. Indeed, despite historical differences, Kurds and Palestinians have in common a history of being low-power groups in asymmetric conflicts. Moreover, they both continue to be denied the formation of an independent state and experience repeated victimization at the hands of high-powered groups. Yet, Norwegians protesting against the maltreatment of Kurds are a rare sight and Norwegian foreign aid to the Kurdish territories is also drastically lower than to Palestine. In 2014 alone, Palestine received 109 million USD from Norway [63], while the *total sum* of all annual aid given to the Kurdish territories between 1995 and 2010 amounts to just 15.5 million USD [67].

Why do Norwegians show far less support for the Kurds than for Palestinians? We expected that Norwegians have substantially more knowledge about the way Palestinians are treated and are more frequently exposed to their suffering by the media. As a result, we expected Norwegian Leftists to show fusion in particular with Palestinians because this group’s perceived oppression clashes ideologically with Leftist beliefs, while this perception may be less pronounced for Kurds. To test this, we randomly assigned participants to a Palestinian or Kurdish condition and predicted that the more Leftist participants were the more they would fuse with Palestinians but not with Kurds. Again, we expected this fusion with the out-group to drive non-normative, extreme protest on the group’s behalf.

### Materials and methods

#### Participants

Through university mailing lists, 215 Norwegian political science students were recruited for a study about “political conflicts” (*M*_*age*_ = 24.99, *SD*_*age*_ = 5.83; women = 59.1%). To ensure that we measured fusion with an ethnic out-group, two participants who either reported to have a Palestinian/Kurdish background or failed to complete this question were removed from the analyses.

#### Procedure

Participants completed the SDO scale from Study 1 (α = .90) and indicated their political orientation on a 10-point scale ranging from 1 (*extremely left-wing*) to 10 (*extremely right-wing*). The latter scale was reversed-scored so that higher values represented a more Leftist political orientation.

Next, participants were randomly assigned to a *Kurdistan* or a *Palestine* condition. This between-subjects design was chosen instead of a within-subjects design to prevent participants from recognizing the comparative purpose of the study and to match their responses towards both groups. They then completed measures from the first study of: fusion with the out-group (α = .88), activist identity (α = .91), political efficacy (α = .92), anger (α = .92), perceived competence (due to an data error, the competence item was only presented to 158 participants), normative protest intentions, and extreme protest, *r*(211) = .44, *p* < .001. Importantly, dependent on condition, the measures were framed towards either Kurds/Kurdistan or Palestinians/Palestine.

Lastly, to test whether participants indeed were more knowledgeable about, and exposed to, the treatment of the Palestinians, they reported their knowledge about the respective conflict on a scale ranging from 1 (*no knowledge at all*) to 10 (*very much knowledge*) and completed five items measuring media exposure frequency to the respective out-groups’ maltreatment (e.g., “How often have you seen [dependent on condition: Palestinians/Kurds] being maltreated on the news during the last year?”; α = .95) ranging from 1 (*never*) to 7 (*very often*).

### Results

In both conditions, participants indicated being relatively Left-wing and no differences between the conditions were observed on this variable (see Table 2). As expected, participants had more knowledge about the Palestinian conflict than about the Kurdish conflict, and had seen more media coverage showing the maltreatment of Palestinians than the maltreatment of Kurds. Moreover, participants in the Palestine condition were more fused with the out-group, had a stronger activist identity and showed more anger and normative protest intentions than those in the Kurdistan condition did (see Table 2).

**Table 2:** Between-group differences for the framing manipulation in Study 2 are displayed.

	Kurdistan Frame		Palestine Frame				
Variable	*M* [95% CI]	*SE*	*M* [95% CI]	*SE*	*F*		η_p_^*2*^
Leftist Orientation	6.83 [6.49, 7.17]	.17	6.97 [6.62, 7.32]	.18	.32		−
Fusion with Out-Group	1.26 [1.17, 1.35]	.04	1.63 [1.47, 1.80]	.08	16.41	***	.07
Activist Identity	1.55 [1.40, 1.70]	.08	2.15 [1.88, 2.38]	.13	15.63	***	.07
Knowledge about the Conflict	5.14 [4.66, 5.62]	.24	6.72 [6.29, 7.15]	.22	23.71	***	.10
Maltreatment Exposure Media	3.69 [3.37, 3.97]	.15	4.35 [4.05, 4.66]	.15	10.22	**	.05
Perceived Competence	6.42 [5.90, 6.94]	.26	6.68 [6.15, 7.20]	.27	.46		−
Anger	4.93 [4.65, 5.22]	.14	5.52 [5.25, 5.80]	.14	8.73	**	.04
Perceived Efficacy	3.43 [3.19, 3.72]	.13	3.52 [3.26, 3.78]	.13	.10		−
Normative Protest Intentions	1.61 [1.37, 1.86]	.13	2.18 [1.82, 2.54]	.18	6.55	*	.03
Extreme Protest Intentions	1.55 [1.34, 1.76]	.10	1.72 [1.49, 1.95]	.12	1.16		−

Note.

*p < .05.

**p < .01.

***p < .001.

#### Moderation analyses

**Fig 2:**
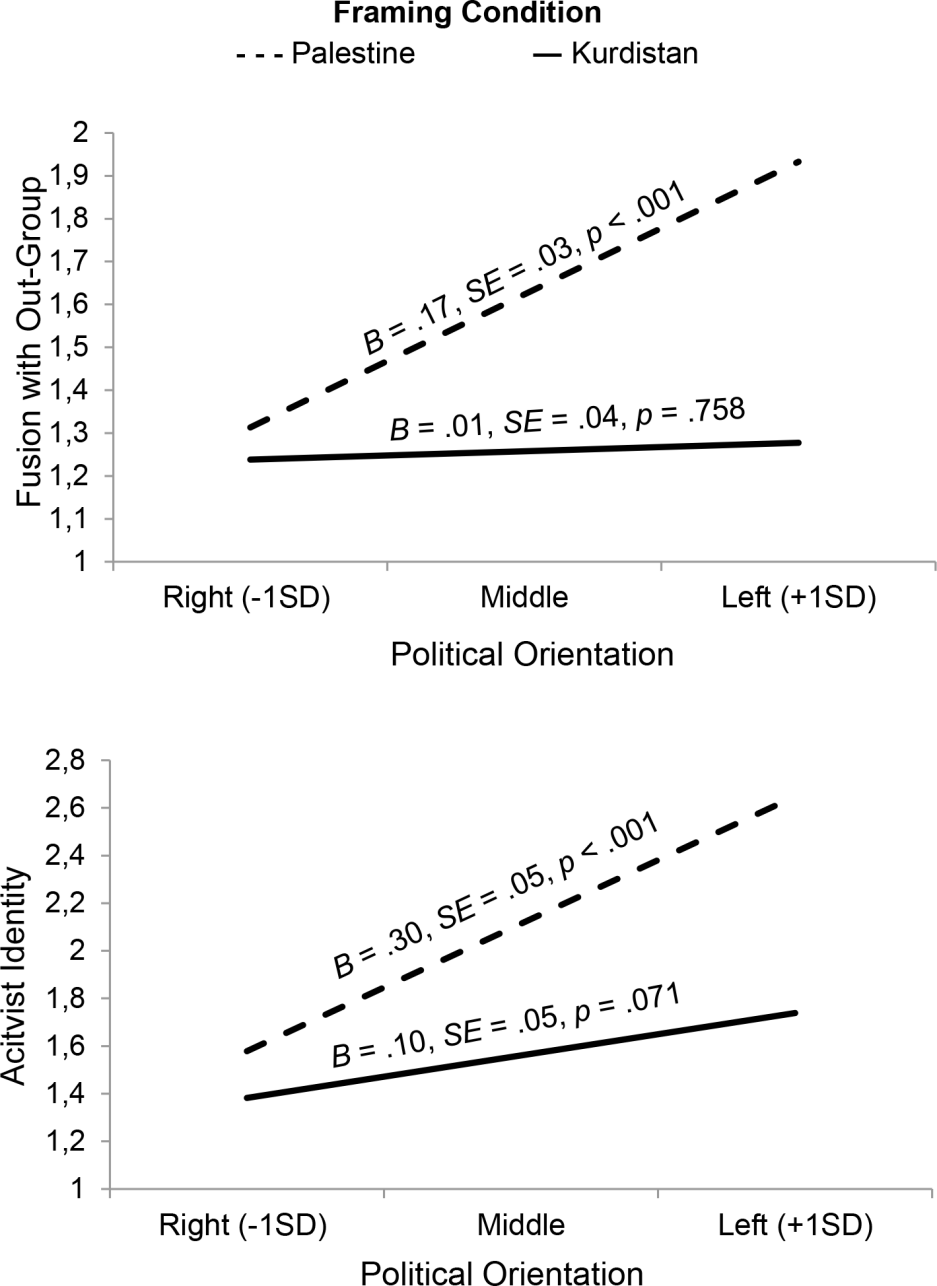
The more Leftist participants’ political orientation was, the more they showed fusion with the out-group and activist identity in terms of Palestine, but not in terms of Kurdistan in Study 2.

As expected, the effects of Leftist political orientation on fusion with the out-group and activist identity were moderated by the framing manipulation. In a first regression with fusion with the out-group as dependent variable, *F*(3, 209) = 14.37, *p* < .001, the framing dummy variable (coded as 0 = Kurdistan, 1 = Palestine; β = .26, *p* < .001, *B* = .37, 95% CI [.19, .54]), the leftist political orientation variable (β = .23, *p* < .001, *B* = .09, 95% CI [.04, .14]) and the interaction between both (β = .21, *p* = .001, *B* = .16, 95% CI [.06, .26]) were significant. Simple slopes showed that leftist political orientation was positively related to fusion with the out-group in the Palestine condition, but not in the Kurdistan condition (see Fig 2). Given that the skewness and kurtosis values of the identity fusion variable (skew = 2.73, kurtosis = 10.23) exceeded the cutoff of +/- 2 and 7 respectively, indicating non-normality [68], we conducted the same analysis after log-transforming the variable (skew = 1.41, kurtosis = 1.59). The same pattern of results was observed applying this transformation.

Also in a second regression, with activist identity as dependent variable, *F*(3, 209) = 17.48, *p* < .001, the experimental dummy variable (β = .25, *p* < .001, *B* = .55, 95% CI [.28, .82]), the Leftist political orientation variable (β = .32, *p* < .001, *B* = .20, 95% CI [.12, .27]) and the interaction between both were significant (β = .16, *p* = .010, *B* = .20, 95% CI [.05, .35]). Here, simple slopes showed that Leftist political orientation was related to more activist identity in the Palestine condition, but not in the Kurdistan condition (see Fig 2). Given that the activist identity variable was only moderately skewed (skew = 1.47, kurtosis = 1.52), the model was not re-estimated log-transforming the variable.

Due to the close relationships of political orientation and SDO in previous research (e.g., [69]) and the present study (see Table 3), we also tested whether the framing manipulation would moderate the effects of SDO on fusion with the out-group and activist identity. However, the interaction terms were non-significant (*ps* > .338).

**Table 3:** Correlations between variables in Study 2 across conditions are displayed.

	2.		3.		4.		5.		6.		7.		8.		9.		10.	
1. Fusion with Out-Group	.64	***	-.05		.25	***	.17	*	.06		.29	***	.22	**	.51	***	.47	***
2. Activist Identity			-.21	**	.34	***	.27	**	.13		.44	**	.28	***	.64	***	.34	***
3. SDO					-.55	***	-.36	***	.03		-.43	***	-.38	***	-.20	**	.00	
4. Leftist Political Orientation							.21	**	.03		.45	***	.36	***	.29	***	.13	
5. Perceived Competence									.14	*	.45	***	.30	***	.25	**	.15	
6. Media Exposure											.26	***	.12		.12		.02	
7. Anger													.36	***	.35	***	.19	**
8. Efficacy															.35	***	.18	*
9. Normative Protest																	.27	***
10. Extreme Protest																		

Note.

*p < .05.

**p < .01.

***p < .001.

#### Moderated mediation model

Given that SDO did not predict any form of solidary action in Study 1, we first ran two regression models in which normative, *F*(3, 209) = 54.29, *p* < .001, and extreme protest, *F*(3, 209) = 19.85, *p* < .001, were regressed on SDO, fusion with the out-group and activist identity to test whether we should include SDO in the full model. Again, however, SDO had unique predictive power in neither model (*ps* > .132), even when we calculated separate dominance (*ps* > .232) and anti-egalitarianism scores (*ps* > .410). This pattern remained the same when we log-transformed the normative protest (before transformation: skew = 2.05, kurtosis = 3.72; after transformation: skew = 1.26, kurtosis = .22) and extreme protest (before transformation: skew = 2.46, kurtosis = 6.65; after transformation: skew = 1.37, kurtosis = .89) variables. Thus, we did not include SDO in the path model.

**Fig 3:**
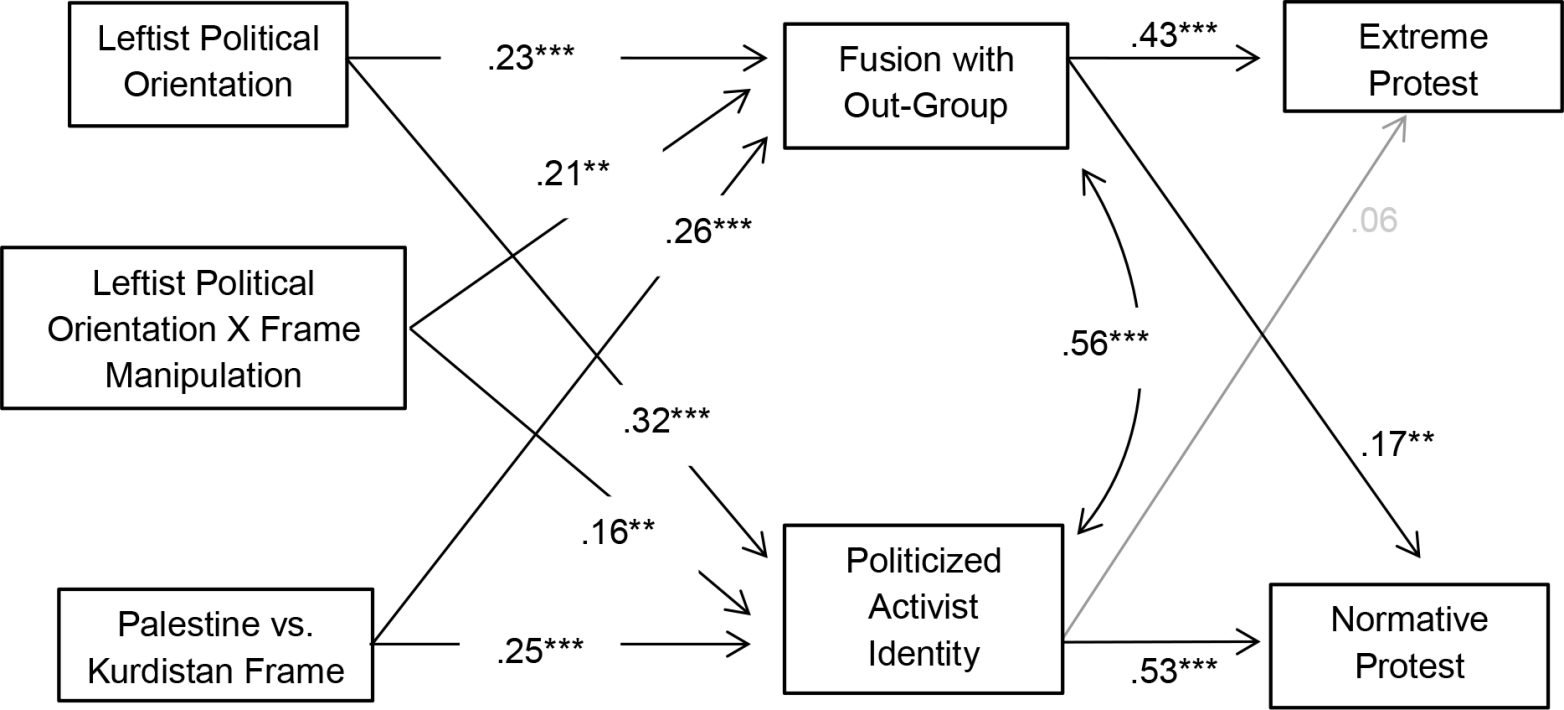
Moderated mediation model in Study 2 is displayed. *p < .05, **p < .01, ***p < .001. Non-significant paths are displayed in grey.

We first tested a basic model in which the experimental framing condition, Leftist political orientation and the interaction between both predicted higher levels of out-group fusion and activist identity, which, in turn, both predicted normative and extreme protest (see Fig 3). In this well-fitting model, χ^2^(*df* = 10, *N* = 213) = 6.15, *p* = .803, *RMSEA* < .001, *CFI* = 1.00, *RMR* = .05, Leftist political orientation, the framing dummy variable and the interaction between both predicted higher levels of fusion with the out-group and activist identity. Fusion with the out-group, in turn, led to more extreme protest and slightly more normative protest intentions, while activist identity led to more normative protest intentions but was unrelated to extreme protest (see Fig 3).

In the Palestine condition, bootstrapping showed that Leftist political orientation indirectly predicted higher levels of extreme protest, as mediated by more fusion with the out-group (β = .21, 95% CI [.07, .39], *p* = .001) but had no indirect effects on normative protest (*p* = .303). In contrast, Leftist political orientation indirectly predicted more normative protest (β = .24, 95% CI [.11, .38], *p* < .001) but not extreme protest for Palestine (*p* = .571) when mediated by higher activist identity.

In the Kurdistan condition, Leftist political orientation had a positive and indirect effect on normative protest that was mediated by stronger activist identity (β = .10, 95% CI [.02, .21], *p* = .009). All other indirect paths were non-significant (*ps >*.386).

To rule out the possibility that what accounts for the effects is mere knowledge about the conflict rather than fusion with the out-group, we first estimated an equivalent path model in which we replaced the fusion variable with the knowledge variable, χ^2^(*df* = 10, *N* = 213) = 9.03, *p* = .526, *RMSEA* < .001, *CFI* = 1.00, *RMR* = .06. In this model, knowledge predicted slightly *less* extreme protest, β = -.14, *p* = .037, but had no effect on normative protest (see S2 Fig in the supplementary materials for the full model). Moreover, no interactive effects of the experimental condition and political orientation on knowledge were observed in the first place. Only the path from the experimental condition on knowledge was significant, indicating that participants had more knowledge about the Israeli-Palestinian conflict than about the Kurdish conflict. Second, we ran a model including the fusion with the out-group variable and controlling for the knowledge variable, χ^2^(*df* = 10, *N* = 213) = 5.29, *p* = .871, *RMSEA* < .001, *CFI* = 1.00, *RMR* = .45. Results showed that effects of fusion with the out-group remained the same when controlling for knowledge (see S3 Fig in the supplementary materials for the full model).

#### Additional analyses

We also tested an extended model to control for anger and political efficacy by letting them mediate the effects of activist identity on solidary action. In the model, χ^2^ (*df* = 28, *N* = 213) = 66.73, *p* < .001, *RMSEA* = .081, *CFI* = .927, *RMR* = .16, efficacy partially mediated the effect of activist identity on normative protest intentions − indirect effect: β = .05, *SE* = .01, 95% CI [.014, .097], *p* = .002 − while the indirect effects mediated by fusion with the out-group remained the same as in the previous model.

### Preliminary discussion

As expected, participants showed more fusion with Palestinians than Kurds. Our results suggested that the different degrees of fusion with Kurds and Palestinians were due to political orientation having distinct effects on both out-groups. Although participants in both conditions had equally Left-wing political views, this orientation only resulted in more fusion with Palestinians. We suspect that this is because, in Norway, the perceived treatment of Palestinians more clearly clashes ideologically with Leftist political beliefs. This interpretation is consistent with our findings that participants reported being substantially more knowledgeable of the Palestinian conflict and also more exposed to the maltreatment of Palestinians via the news media. Finally, Leftist political orientation was related to a higher activist identity but only predicted normative forms of protest. Fusion with the out-group, however, predicted extreme non-normative protest. This suggests that fusion may not only be a more potent predictor of extreme behavior than social identity when it comes to in-groups but also in terms of extreme solidary action for groups one is not part of.

While this study demonstrated that the political orientation–fusion pathway can explain why people are willing to go to extreme means to support one out-group but not another, the next study aimed to further uncover our proposed underlying mechanism. Specifically, we sought to causally test our proposal that the reason that Leftists do not fuse with Kurds is that their situation is not violating core Leftist values because it is not perceived as an asymmetrical conflict.

## Study 3

To provide direct support for our argument that fusion with out-groups occurs when the group’s treatment clashes with one’s political views, here, we directly manipulate perceptions of the out-group’s situation. Specifically, because social justice [39, 40], resistance to oppression [49, 53, 70] and the right of self-determination [41, 71] are core Leftist values, we experimentally framed the Kurds as either being engaged in a symmetrical war or as being the victims of oppressive occupation, and predicted that the latter would increase fusion with and, subsequently, lead to more willingness to engage in non-normative extreme protests on behalf of Kurds.

### Materials and methods

#### Participants

A total of 234 European-Americans (*M*_*age*_ = 36.13, *SD* = 11.62; men: 45.7%) participated through Amazon MTurk in a study on “social issues.” All participants were paid $2.50 for participation.

#### Procedure

Participants first completed the Leftist political orientation scale and then indicated how knowledgeable they were about the Kurdish conflict on the same scales as in the previous study. We then manipulated the out-group’s situation by randomly assigning participants to a *war* condition, an *oppressive occupation* condition or a *control* condition. In each condition, participants were instructed to read a text allegedly written by a Kurdish individual. In the war and occupation conditions, the text comprised a narrative describing Kurds living under war or under oppressive occupation yet the wording was otherwise identical. The text in the *war* condition was:

"When I came into the world and first opened my eyes, we Kurds were fighting in a war.

For as long as I know, our people have been at on-going war with the Turks, the Arabs and the Persians.

More than 100 000 of our civilians, including many children and women, have become victim of this war, and every day brings more tragedy.

My only son was shot in front of my eyes because he was fighting the enemy soldiers. There is hardly a day that I don’t stare out of the window thinking about him…and how things would have been different without this war.

Living under war has left a mark on our people’s development as a sovereign nation. The war has not only caused a lot of suffering and families being teared apart, but also negatively affected our language and cultural heritage–in short, undermining the very essence of who we are.

I often dream and long for a war-free Kurdistan, with no fighting, where every Kurd could have the chance to live in peace and have the possibility to live her/his life with dignity."

The text in the *oppressive occupation* condition was:

"When I came into the world and first opened my eyes, we Kurds were under occupation.

For as long as I know, our people have been systematically oppressed by the Turks, the Arabs and the Persians.

More than 100 000 of our civilians, including many children and women, have become victim of this occupation, and every day brings more tragedy.

My only son was shot in front of my eyes because he resisted the occupying soldiers. There is hardly a day that I don’t stare out of the window thinking about him…and how things would have been different without this occupation.

Living under occupation has left a mark on our people’s development as a free nation. The occupying powers have not only caused a lot of suffering and families being teared apart, but also negatively affected our language and cultural heritage–in short, undermining the very essence of who we are.

I often dream and long for a free Kurdistan under no occupation, where every Kurd could have the chance to be free and have the possibility to live her/his life with dignity."

In the *control* condition, participants read a text about Kurds’ ethnic origins and language:

"The Kurdish people are an ethnic group that originated in the Middle East. Currently, Kurds are living in parts of Turkey, several Arab countries and Iran. The Kurds historically inhabited the regions surrounding the Zagros Mountains. The area is often referred to as Kurdistan.

Many Kurds consider themselves descended from the ancient Medes, and even use a calendar dating from 612 B.C.

The Kurdish languages form a subgroup of the Northwestern Persian languages. Just as the Kurdish people inhabit different countries, the Kurdish language is written in a range of scripts, including the Perso-Arabic alphabet and the Latin alphabet.

Kurdish is an official language of Iraq and a regional language in Iran. Many Kurds are bilingual.

Kurdish culture is a legacy from the various ancient peoples who shaped modern Kurds and their society. As most other Middle Eastern populations, a high degree of mutual influences between the Kurds and their neighboring peoples are apparent."

The three texts were matched in terms of length, number of paragraphs and format. To check the effectiveness of the manipulation, on the next page, participants rated the extent to which they would describe the conflict as a war vs. occupation from 1 (*war*) to 10 (*occupation*). Next, in randomized order, participants completed the fusion with the out-group (α = .96) and the activist identity scales (α = .94), followed by one item measuring normative protest intentions, and two items measuring extreme protest, *r*(232) = .53, *p* < .001, as in the previous studies. Because of the absence of SDO effects in the first two studies, the variable was not measured.

### Results

#### Manipulation check

The manipulation check indicated that the experimental manipulation was successful. In an univariate analysis of variance (ANOVA), the conflict type manipulation predicted perceptions of the conflict being an occupation rather than war, *F*(2, 231) = 17.71, *p* < .001, η_p_^2^ = .13. Post-hoc tests with Bonferroni correction showed that participants in the occupation condition, to significantly larger extent (*ps* < .001), described the conflict as an occupation compared to participants in the control condition (*M* = 7.30, *SE* = .28, 95% CI [6.76, 7.85] vs *M* = 5.64, *SE* = .26, 95% CI [5.13, 6.16]), but especially compared to participants in the war condition (vs. *M* = 5.11, *SE* = .26, 95% CI [4.59, 5.63]). The latter two conditions did not differ significantly from each other (*p* = .467). No unmoderated, main effects were observed on the mediators (i.e., fusion with the out-group: *p* = .970; activist identity: *p* = .623) and dependent variables (i.e., normative protest: *p* = .682; extreme protest: *p* = .167).

#### Moderated mediation model

**Fig 4:**
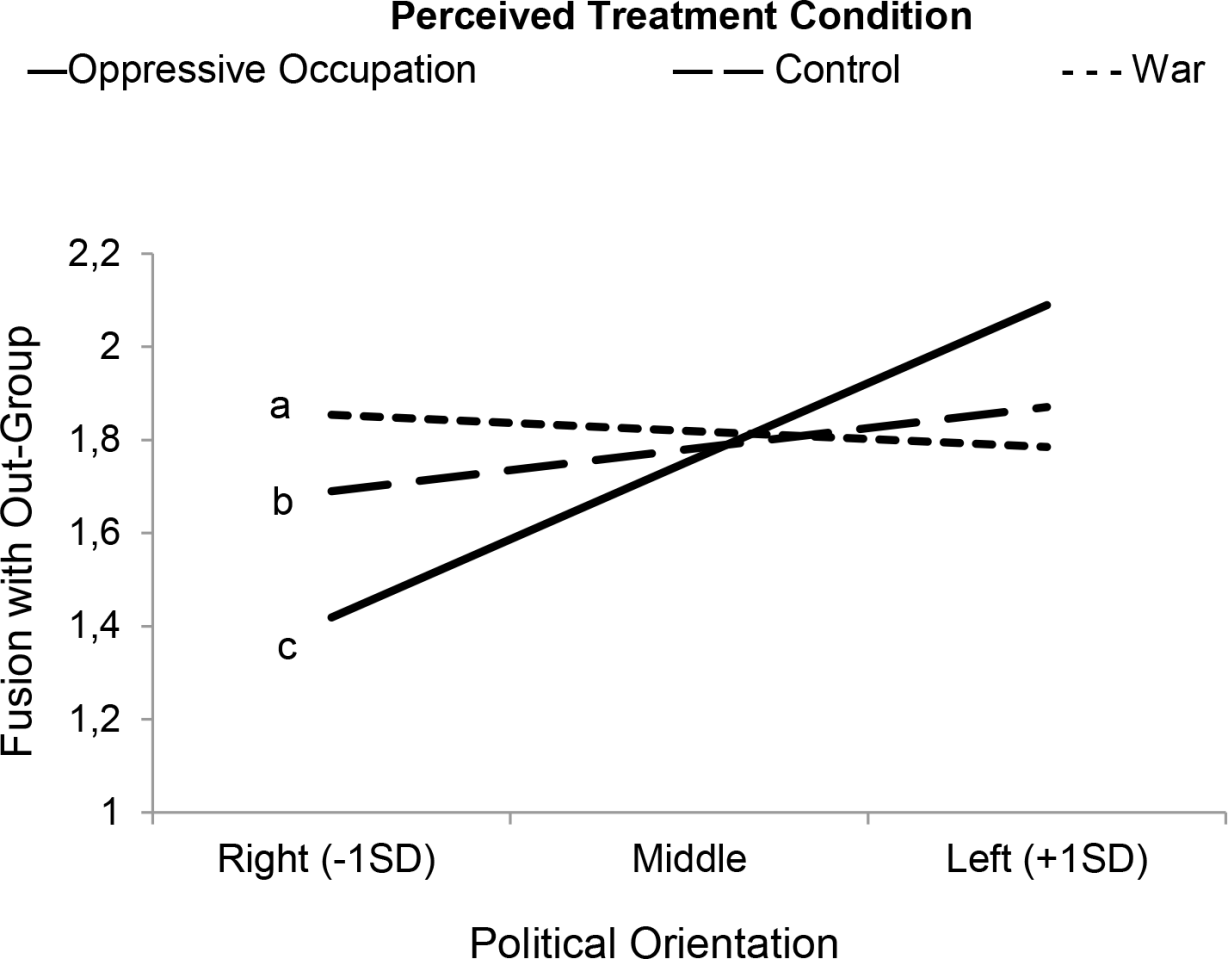
In Study 3, Leftist political orientation predicted more fusion with the Kurdish out-group when they were described as being victims of oppressive occupation but was unrelated to fusion with the out-group in the control and war condition. ^a^*B* = -.01, *SE* = .04, *p* = .760, 95% CI [-.10, .07]; ^b^*B* = .03, *SE* = .05, *p* = .531, 95% CI [-.07, .14]; ^c^*B* = .13, *SE* = .05, *p* = .014, 95% CI [.03, .23].

We set out to estimate first-stage moderated mediation models [72] in which the treatment type manipulation moderated the effects of political orientation on fusion with the out-group which, in turn, was expected to predict higher levels of normative and extreme protest. Before conducting the analyses, we created two dummy variables, one comparing the oppressive occupation prime to the control condition and one comparing it to the war condition. All predictors were mean-centered. As expected, in the moderated regression model with fusion with the out-group as dependent variable, Leftist political orientation significantly interacted with the dummy variable that compared the oppressive occupation with the war prime (see Table 4). As simple slopes indicated, when the Kurds were described as victims of oppressive occupation, Leftist political orientation predicted higher levels of fusion with the out-group, but not when they were described as being part of a war (see Fig 4). While the interaction with the dummy variable comparing the oppressive occupation to the control condition was non-significant, simple slopes showed that political orientation also was unrelated to fusion with the out-group in the control condition. None of the interactions were significant for activist identity (see Table 4). Because the skewness and kurtosis of activist identity (skew = 1.94, kurtosis = 3.64) and fusion with the out-group (skew = 1.75, kurtosis = 2.74) fell within the normal range [68], we did not re-estimate the models with log-transformed variables.

**Table 4:** Moderated regression models with fusion with the out-group and activist identity as dependent variables in Study 3 are displayed.

	Fusion with Out-Group^a^					Activist Identity^b^				
Variable	*B*	*SE*	*p*	*LLCI*	*ULCI*	*B*	*SE*	*p*	*LLCI*	*ULCI*
Leftist Political Orientation	.13	.05	.014	.03	.23	.06	.05	.233	-.04	.17
Occupation vs. Control^1^	.62	.53	.237	-.41	1.66	.03	.54	.952	-1.04	1.11
Occupation vs. War^1^	.97	.47	.041	.04	1.90	.22	.49	.653	-.74	1.18
Leftist Orientation X Occupation vs. Control	-.09	.08	.217	-.24	.055	.02	.08	.757	-.13	.18
Leftist Orientation X Occupation vs. War	-.14	.07	.037	-.27	-.01	-.02	.07	.730	-.16	.11

*Note*. All independent variables were mean centered before the model was estimated.

^a^*F*(5, 228) = 1.33, *p* = .252

^b^*F*(5, 228) = 1.12, *p* = .350.

^1^The occupation condition was coded as 0 and the respective comparative condition as 1.

Given that the mediator variable of fusion with the out-group, in turn, predicted more normative and extreme protest (see Table 5), we used model 7 of the PROCESS macro [73] to test our full moderated mediation model. In these models, political orientation was expected to have indirect effects on normative and extreme protest, mediated by fusion with the out-group, when the Kurds were described as victims of oppressive occupation but not when they were described as part of a war. Because the macro does not provide standardized coefficients and *p* values for indirect effects, we present unstandardized coefficients with upper and lower 95% confidence intervals here. As predicted, bootstrapping showed that leftist political orientation had significant and positive indirect effects on normative (*B* = .08, *SE* = .04, 95% CI [.03, .19]) and extreme protest (*B* = .08, *SE* = .03, 95% CI [.03, .15]) when the Kurds were described as victims of oppressive occupation, while the indirect effects were non-significant in the war condition (normative protest: *B* = -.01, *SE* = .04, 95% CI [-.08, .07]; extreme protest: *B* = -.01, *SE* = .03, 95% CI [-.07, .06]). While the extreme protest variable had acceptable skew (skew = 1.75, kurtosis = 2.87), we re-estimated the indirect effects with a log-transformed normative protest variable as it was positively skewed (before transformation: skew = 3.16, kurtosis = 9.63; after transformation: skew = 2.28, kurtosis = 4.10). Replicating the previous results, the indirect effect on the log-transformed normative protest variables was significant in the occupation condition (*B* = .01, *SE* = .01, 95% CI [.01, .03]) but not in the war condition (*B* = -.001, *SE* = .01, 95% CI [-.01, .01]).

**Table 5:** Correlations between variables in Study 3 across conditions are displayed.

	2.	3.		4.		5.		6.		7.	
1. Leftist Political Orientation	.10	.13	*	.15	*	-.10		.04		.05	
2. Fusion with Out-Group		.78	***	.04		.36	***	.60	***	.57	***
3. Activist Identity				.04		.41	***	.56	***	.52	***
4. Perceptions of Occupation						.19	**	.14	*	.09	
5. Knowledge about Conflict								.40	***	.29	***
6. Normative Protest										.64	***
7. Extreme Protest											

Note.

*p < .05.

**p < .01.

***p < .001.

### Preliminary discussion

As predicted, when the Kurds were described as victims of oppressive occupation rather than as being engaged in symmetrical war, being Leftist resulted in more fusion with Kurds and, consequently, more willingness to engage in non-normative extreme protests on their behalf (and, to a smaller extent, normative protest as well). Hence, we were able to demonstrate that being Leftists makes people fuse only with out-groups that are perceived to be victims of the asymmetrical oppression that clashes with Leftist political ideology. This finding provided critical insight into why Leftists fuse with some out-groups and not others.

## Study 4

So far, we have investigated whether fusion with out-groups can explain a hypothetical willingness to engage in extreme and non-normative activism for out-groups. But, a question that remains is whether our framework can also be applied to those who are *actually* interested in risking their lives for a distant out-group. To test this, we sampled aspiring foreign fighters for the Kurdish People’s Protection Units (i.e., YGP) in their fight against ISIS.

Another goal of this study was to integrate our framework with research suggesting that people feel morally obliged to enforce the kinds of relationships and values they endorse [74]. For instance, in the fight for equality against oppression, people are especially likely to support an out-group when the norm is to feel moral outrage about the group’s maltreatment [51, 75, 76]. Because fused people tend to be especially likely to uphold their moral standards even if their life is at-risk [21], we hypothesized that a Leftist political orientation should predict more fusion with Kurds insofar as people feel morally compelled by their political ideology to support the Kurds’ struggle.

Importantly, because many countries have experienced terror attacks by ISIS and are militarily involved in the fight against them, we also assessed and controlled for whether high fusion with one’s own group would be an alternative motivator to join the YPG. Finally, in this study we aimed to rule out two alternative explanation for why Leftists fuse with Kurds: 1) that they perceive them to have the same political orientation as themselves and, thus, that our results simply reflect fusion with the same political group [8] or 2) because the enemy group (i.e., ISIS) has a divergent political orientation and, thus, that our results simply reflect a desire to fight those with contrasting political views.

### Materials and methods

#### Participants

A total of 83 participants (*M*_*age*_ = 31.6, *SD*_*age*_ = 8.8; men = 96.3%) were recruited for a study on “motivations to join the Kurdish YPG” through Facebook pages for people interested in volunteering as foreign fighters in the Kurdish militia fighting ISIS (e.g., the page “Lions of Rojava”). To be asked to participate, participants had to have made at least one post explicitly requesting information about how to volunteer for the Kurdish militia. They came from a total of 24, predominantly Western, countries and mostly from the U.S. (45.7%), the U.K. (9.9%) and Canada (6.2%). None were from a Middle Eastern country or reported to have Kurdish parents. Of all participants, 9.6% had already joined the YPG. Participants completed the following measures on 7-point Likert scales ranging from 1 (*totally disagree*) to 7 (*totally agree*) unless stated otherwise:

#### Leftist political orientation

On the same scale as in the previous studies, participants indicated their political orientation, which was reversed-scored so that higher values represented more Leftist political orientation.

#### Political moral obligation

On a scale ranging from 1 (*not at all*) to 10 (*to very large extent*), participants completed the question, “To which extent do you think that people with your political orientation have the moral obligation to support the Kurds in their struggle?”

#### Perceived orientation of Kurds and ISIS

On the political orientation scale from the previous studies, participants were asked to rate the political orientation they believed most Kurdish people and most ISIS people had. Responses were reverse-scored.

#### Fusion with the in-group and the out-group

With the same scales as in the previous studies, we measured fusion with Kurds (α = .87) and fusion with participants’ own ethnic in-group (α = .92).

#### Extreme group behavior

Three items (e.g., “I would fight someone physically threatening a Kurd”) adopted from Swann et al. [23] were used to measure extreme group behavior (α = .65).

#### Self-sacrifice

Two items (e.g., “I would sacrifice my life if it saved a Kurd’s life”), also adopted from Swann et al. [23] measured willingness to self-sacrifice, *r*(77) = .58, *p* < .001.

#### Likelihood to join YPG

On a sliding-response scale ranging from 0% to 100%, participants indicated how likely it was that they would join the YPG.

### Results

Participants reported more fusion with Kurds than with their own ethnic in-group (see Table 6). They also perceived Kurds to have a more Leftist political orientation than ISIS.

**Table 6:** Means, Standard Deviations and Correlations between variables in Study 4 are displayed.

	*M* (*SD*)	2.	3.		4.		5.	6.		7.		8.		9.	
1. Leftist Orientation	4.90 (3.15)	.01	.51	***	-.45	***	.12		-.24	*	.14	-.14		.01	
2. Moral Obligation	8.28 (1.97)		.04		.01		.12		.03	.34	**	.13		.16	
3. Perc. Kurdish Orientation^1^	4.98 (3.10)				-.53	***	-.06		-.10	.21	^†^	-.10		-.01	
4. Perc. ISIS Orientation^1^	2.29 (3.26)						-.04	.12		-.10		.16		.05	
5. Fusion with the Out-Group^2^	5.36 (1.32)							.44	***	.30	**	.43	***	.33	**
6. In-Group Fusion^2^	4.89 (1.64)									.13		.28	*	.08	
7. Extreme group behavior	6.08 (1.04)											.26	*	.61	***
8. Likelihood to Join YPG	86.81 (19.02)													.40	***
9. Willing to Self-Sacrifice	6.32 (1.05)														

*Note*. Higher scores mean more leftist political orientation on all political orientation variables. ^1^*t*(79) = 4.38, *p* < .001.

^2^*t*(81) = 2.63, *p* = .010.

^†^*p* = .066.

*p < .05.

**p < .01.

***p < .001.

#### Moderation analyses

**Fig 5:**
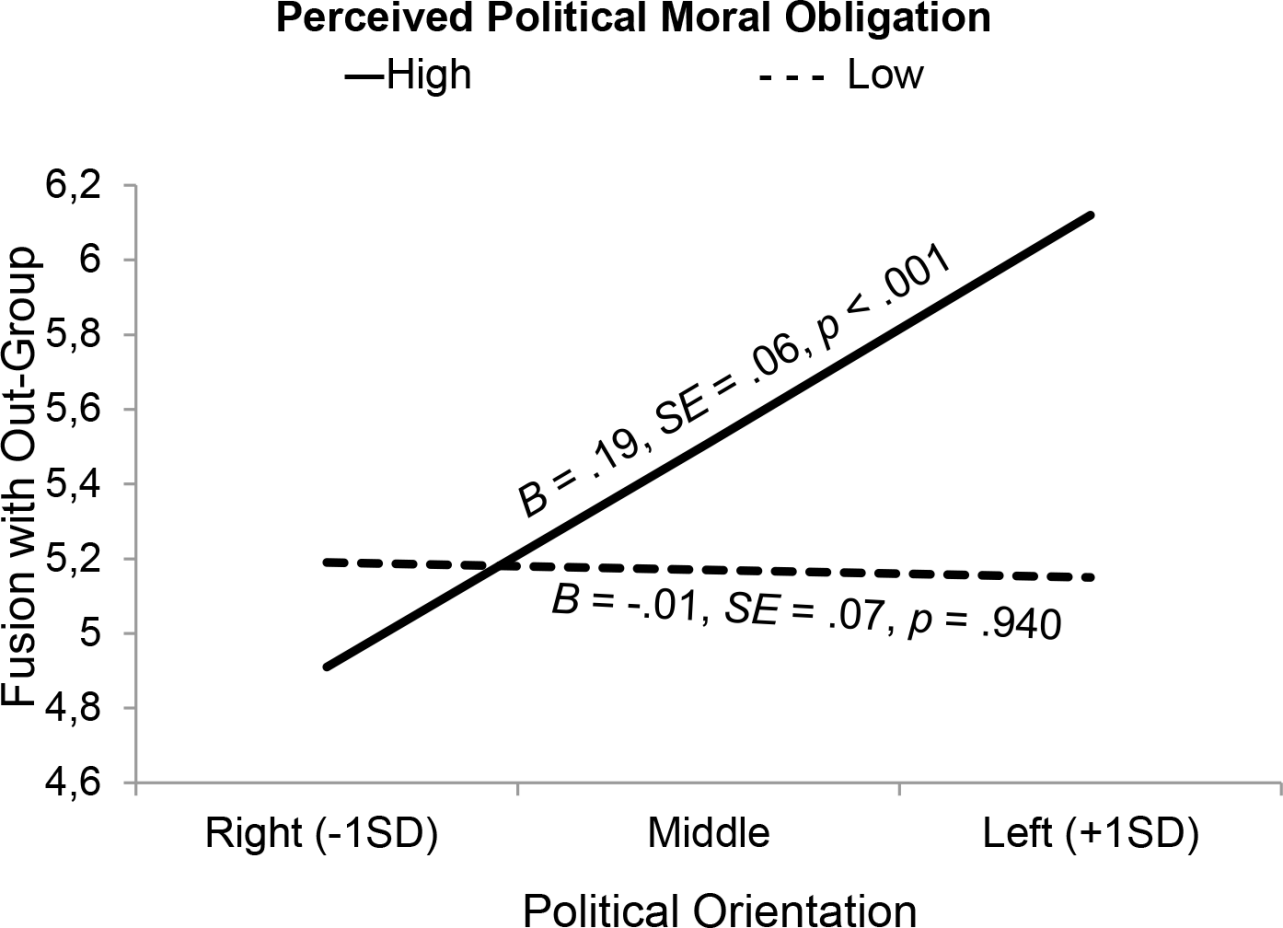
In Study 4, Leftist political orientation predicted more fusion with the Kurdish out-group when aspiring foreign fighters experienced that they had a high moral obligation to support the Kurds.

We ran a regression model to test whether moral obligation, perceived Kurdish political orientation or perceived ISIS political orientation moderated the effects of participants’ own Leftist political orientation on fusion with Kurds. Here, we also controlled for identity fusion with one’s own racial/ethnic group as it was positively related to fusion with the out-group (see Table 6). The predicted interaction between one’s own Leftist political orientation and moral obligation was significant (see Table 7). Simple slopes showed that when participants experienced a high political moral obligation to support the Kurds, Leftist political orientation predicted more fusion with the out-group but not when moral obligation was low (see Fig 5). This model was not re-estimated with a log-transformed fusion with the out-group variable because it was relatively normally distributed (skew = -.74, kurtosis = -.12).

**Table 7:** Moderated regression models for fusion with the out-group in Study 4 is displayed.

		Fusion with Kurds^a^				
Variable	β	*B*	*SE*	*p*	*LLCI*	*ULCI*
Fusion with Own Group	.53	.43	.08	< .001	.27	.58
Leftist Orientation	.24	.10	.05	.044	.003	.20
Perceived Kurdish Political Orientation	-.24	-.11	.05	.041	-.21	-.004
Perceived ISIS Political Orientation	-.20	-.08	.06	.151	-.20	.03
Perceived Moral Obligation	.14	.09	.07	.163	-.04	.22
Leftist Orientation X Perceived Kurdish Orientation	.12	.02	.02	.303	-.02	.05
Leftist Orientation X Perceived ISIS Orientation	-.04	-.01	.02	.790	-.039	.029
Leftist Orientation X Perceived Moral Obligation	.27	.05	.02	.008	.015	.092

*Note*. All independent variables were mean centered before the model was estimated.

^a^*F*(8, 70) = 5.84, *p* < .001.

Confidence intervals are unstandardized.

#### Moderated mediation model

Given that fusion with the out-group was associated with higher levels of extreme group behavior, self-sacrifice and likelihood to join the YPG, we estimated moderated mediation models using model 7 of the Process macro based on the model estimated above. Bootstrapping showed that Leftist political orientation indirectly led to a higher likelihood to join the YPG, more extreme group behavior, and willingness to self-sacrifice because it increased fusion with the out-group when participants experienced high moral obligation, but not when they experienced low moral obligation (see Table 8). While the skewness and kurtosis of the likelihood to join the YPG (skew = -1.97, kurtosis = 4.65) and extreme group behavior (skew = -1.79, kurtosis = 4.31) variables fell within the suggested cutoffs [68], the values of the willingness to sacrifice variable exceeded this cutoff (skew = -2.67, kurtosis = 9.15). Hence, we log-transformed and reversed the variable (skew = -1.09, kurtosis = .77) and re-estimated the indirect effects. Replicating the previous results, Leftist political orientation had an indirect effect on willingness to self-sacrifice when perceived moral obligation was high (*B* = .01, *SE* = .01, 95% CI [.001, .03]), but not when it was low (*B* = -.003, *SE* = .01, 95% CI [-.02, .01]).

**Table 8:** Indirect effects of Leftist orientation on dependent variables mediated by fusion with the out-group at different levels of the moderator perceived moral obligation in Study 4.

Dependent Variable	Level on Moderator (Moral Obligation)			95% CI	
		*B*	*SE*	Lower	Upper
Likelihood to Join YPG	Low	-.01	.61	-1.279	1.182
	High	1.24	.62	.245	2.739
Extreme Group Behavior	Low	-.01	.03	-.066	.042
	High	.04	.03	.005	.107
Willingness to Self-sacrifice	Low	-.01	.03	-.081	.053
	High	.05	.03	.005	.131

*Note*. Estimates are based on bias-corrected bootstrapping with 5000 random re-samples.

### Preliminary discussion

Using a sample of aspiring foreign fighters, here we successfully replicated our framework and, thus, provide crucial ecological validity to our paradigm. Strikingly, participants in this unique sample were more fused with the Kurdish out-group than their own ethnic in-groups. This finding is similar to research on in-group fusion by Whitehouse et al. [19] showing that about one half of a group of Libyan rebels were more fused with their battalions than with their families. Moreover, we found that Leftist were more fused with Kurds, and thus more willing to fight on their behalf, when they felt that their political orientation morally compelled them to support the Kurdish struggle. This further supports and extends our overall argument that people fuse with out-groups when the way this group is treated (e.g., being oppressed) clashes with one’s core political worldview and ideology. In contrast, personally having a political orientation that overlaps with that of the Kurds or is in opposition to that of ISIS were both unrelated to such willingness to fight on their behalf, ruling out these two alternative mechanisms. In other words, it is not simply one’s degree of perceived political similarity towards the out-group, or one’s dissimilarity with the out-group’s enemies, that drives the political orientation–fusion with the out-group pathway we have demonstrated.

## General discussion

From individuals aspiring to become foreign fighters in the Kurdish militia’s struggle against ISIS to Americans risking their lives for Palestinian rights, there are many examples of those willing to engage in extreme and risky support for out-groups based on their personal or political orientations. Building on previous research that demonstrated that feelings of “oneness” with distant in-groups at various levels of abstraction (referred to as "extended identity fusion" [7]) can lead people to engage in extreme solidary efforts, the present paper demonstrated that the process of fusing with groups one originally did not belong to may even motivate extreme efforts in support of *out-groups*. Importantly, we also demonstrated that perceiving the out-group’s treatment as violating one’s own political ideology and morals motivates such fusion with the out-group.

Across various cultural contexts (e.g., U.S., Norway), populations (i.e., general population and aspiring foreign fighters) and conflicts (Israel-Palestine, Kurdish, war against ISIS), four studies supported this general pattern of findings. Specifically, in Study 1, the more politically Leftist individuals were, the more fused they were with the Palestinian out-group, which led to increased willingness to engage in extreme protest on their behalf. In Study 2, Leftists demonstrated this pattern more with Palestinians than Kurds. In Study 3, we directly investigated *why* this might occur, hypothesizing that the reason is that Palestinians more than Kurds are perceived as a fitting case for Leftist ideological support for the downtrodden of the world. Supporting this prediction, we found that experimentally framing the treatment of Kurds as clashing with Leftist ideology (by describing them as victims of oppressive occupation) increased fusion with the out-group and, in turn, willingness to engage in extreme protest on their behalf among Leftists. Finally, in Study 4, Western Leftists aspiring to be foreign fighters for the Kurdish YPG fused more with Kurds when feeling morally compelled by their political orientation which, in turn, resulted in stronger intentions to fight against ISIS.

The main theoretical contributions of this research are two-fold. In an ever more globalized world, our research provides the first empirical demonstration of the role of fusion in explaining extreme solidary action on behalf of distant out-groups. Moreover, it did so in a variety of contexts that allowed for a clear operationalization of fusion with an out-group. Specifically, participants in our studies shared no racial/ethnic group membership with the respective out-groups and, except for Study 4, were not engaged in the same conflict as the out-group. In accordance with previous research on fusion with one’s own group (e.g., [20]), we found that fusion with an out-group had its effects primarily on non-normative extreme protests. Indeed, this was the case even when controlling for a range of alternative variables stemming from the social identity and social dominance approaches to collective action [48, 50]. Since we observed relatively consistent effects across contexts and when controlling for a range of alternative predictors, we suggest that the process of fusion with out-groups should be integrated into models of solidary collective action, particularly when the aim is to explain *extreme* support of out-groups.

The second main contribution of this research is that it highlighted one potent mechanism explaining why and under which circumstances people fuse with out-groups. Specifically, it showed that fusion with a former out-group occurs when the way the out-group is treated clashes with people’s own political orientation and ideology. This finding is consistent with previous research demonstrating that individuals may fuse with their in-groups especially when they perceive sacred values to be threatened [12, 13].

Both of these theoretical contributions open up several avenues for future research in the fields of intergroup relations. For instance, a vital next step could be to explore fusion with out-groups among Right-wing individuals. While our results could be interpreted as Right-wing individuals showing especially low degrees of fusion with out-groups [77], it is entirely possible that, while de-fusing from low-power groups (i.e., the oppressed), they simultaneously fuse with high-power out-groups (e.g., the oppressors or dominators) involved in the same political conflicts. Retrospectively, this might explain why thousands of non-German (and “non-Aryan”) volunteers joined, and often lost their lives for, the Nazi’s Waffen SS in the Second World War. Future studies should test this proposal with contemporary scenarios. For instance, it could be tested whether Right-wing individuals would fuse with ruling, authoritarian political out-groups when subordinate groups challenge their position and whether the degree of existing societal inequality or oppression amplifies the potential for violent retaliation in this case (cf. [78]) Moreover, following research and theorizing on the role of sacred values [12–14], such future research may profitably replicate our paradigm by directly assessing the extent to which participants endorse a certain set of values rather than, as we did, measuring their broader political orientation.

We also encourage future research to use more comprehensive multi-item scales to measure extreme group behavior. We used two-item scales to measure extreme protest in Studies 1 through 3, and two- and three-item scales to measure self-sacrifice and extreme group behavior in Study 3, all of which were adopted from previous social identity [60, 61] and identity fusion research [23]. The correlations between the two items of the extreme protest scale used in Studies 1 through 3 were moderately strong (*rs* = .44 to .53). This raises potential questions regarding the unidimensionality of the scale. While the first item measured a personal willingness to engage in violent protest, the second item captured whether one saw violent protest as “the only mean” to achieve social change. Despite the fact that these items have been assumed to measure the same construct in previous research, it is possible that each captured somewhat different sub-facets of violent protest. The use of more elaborative multi-item scales with higher reliability may hence be advisable for future research. Moreover, several of the variables, and here in particular the normative and extreme protest measures, tended to be skewed. This was not very surprising as the phenomena under investigation would be expected to be relatively uncommon in normal populations. Although results held when log-transforming skewed variables so that they met assumptions of normality, future research may aim to gather data from more extreme groups (such as the one in Study 4), in which a general willingness to engage in extreme group behavior is higher.

Another important avenue for future work is to identify mediators of the effects of fusion with out-groups on non-normative extreme out-group support. In contrast to previous work (i.e., [79]), we found that none of the classic social identity mediators accounted for the observed effects of fusion with out-groups. Yet, future research might examine recently proposed social identity factors such as the emotion contempt which may predict non-normative social action to larger extent than anger [80]. We also note that it could have been meaningful to include a multi-factorial measure of social identity, including the solidarity facet, because in previous research it predicted extreme group behavior, albeit to significantly less extent than identity fusion [23]. However, additional alternative mediators that are not part of the social identity framework were likely at play in the present research. As suggested by previous work on identity fusion with one’s own group [7, 20], we suspect that feelings of invulnerability or high risk tolerance may also mediate effects of fusion with out-groups on extreme out-group support. Indeed, such findings would explain why the fused aspiring foreign fighters in our last study were dedicated to join the fight against ISIS despite extensive publicly-available footage showing the brutal executions of their antagonists. Future research may also benefit from comparing antecedents and outcomes of fusion with out-groups to those of in-group fusion. For instance, one might expect that individuals who believe that groups share an inherent essence (as in essentialist beliefs [81]) may be *more* likely to fuse with in-groups, as suggested by previous research [20, 82], but *less* likely to fuse with out-groups. In contrast, participant who perceive some higher-order shared group membership [83, 84], such as a common humanity [85], may show more fusion with out-groups.

Furthermore, comparing the outcomes of in-group fusion and fusion with out-groups in zero-sum scenarios between both groups, including moral dilemmas, may be informative. In previous research, in-group fusion predicted more self-sacrifice to save in-group members at various levels of abstraction but not self-sacrifice for out-group members [10]. How would individuals who are fused with both groups respond to these dilemmas? And, would individuals who show stronger fusion with the out-group than the in-group–as was the case for most participants in the last study–sacrifice in-group interests or even in-group members if it saved out-group members? Such perspectives may also help understanding recent societal events such as White Americans risking their lives to rally against White Supremacy in Charlottesville, Virginia. Research could test whether such protesters were more fused with ethnic out-groups (that are the enemy of White supremacists) than their White ethnic in-group.

We acknowledge that it is possible that some participants may have shared some group memberships with Palestinians/Kurds on dimensions other than race/ethnicity. For instance, we did not assess participants’ religious group. It may be that some of the participants had an Islamic belief in common with the out-groups. In light of previous research demonstrating the role of fusion with religious groups for extreme group behavior [32], it is therefore possible that Muslim identification or fusion also was at play. However, Kurds and Palestinians both are predominantly Muslim as are the groups with which the Kurds (but not Palestinians) are in conflict with, which to some extent attenuates concerns about such an alternative predictor. It is also important to note that Muslims are a very small minority among White Americans and Norwegians, so that such a process would have applied to very few of the participants in most of the studies. Similarly, we did not measure whether participants had out-group friends, which could have fostered a fictional sense of kinship. Future research should thus measure and control for such additional variables to further establish the unique effects of fusion with out-groups.

Relatedly, one may also ask the question whether fusion with an ethnic out-group may be more accurately described as in-group fusion. As described in the introduction, we used the term “out-group” to denote a group that participants did not belong to in terms of their ethnic background *before* fusing with it. Indeed, when asked about their ethnic group, all participants whose data was analyzed indicated to belong to other ethnic groups than Kurds/Palestinians. Hence, at least in terms of actual kinship, both can be seen as out-groups. Also, Kurds and Palestinians constitute national out-groups, which is a strength compared to previous identity fusion research in which participants ethnicity often was nested within, or covaried with, the group they fused with. To be sure, following a social identity perspective, for participants who fused with Palestinians or Kurds, the respective group likely formed a part of their self-concept. As such, once individuals fuse with the out-group, obviously they may come to regard it as their in-group, and this group may even appear more relevant to them than pre-existing ethnic categories (as arguably was the case in the last study). The point of the present research is that it is indeed possible to feel a sense of visceral oneness with ethnic groups that clearly share no biological ancestry with oneself: fusion processes nevertheless account for the solidary action one is willing to take on their behalf. Crucially, what appears to lead to this stage of extended fusion is the relational perception that the way the out-group is treated violates one’s political ideology.

## Conclusion

In sum, the present research extended the concept of identity fusion by showing that not only does it explain extreme behavior for one’s own group, but also for what is initially one’s ethnic out-group with which one shares no biological ancestry. This fusing with out-groups appears to be driven and motivated, at least in part, by perceiving them as being treated in a way that clashes with one’s own political beliefs.

## Supporting information

S1 Fig Reversed mediation model for Study 1. Non-significant paths are displayed in grey. *p < .05, **p < .01, ***p < .001.
(TIF)

S2 Fig Moderated mediation model for Study 2, in which fusion with the out-group is replaced with knowledge about the conflict. Non-significant paths are displayed in grey. *p < .05, **p < .01, ***p < .001.
(TIF)

S3 Fig Moderated mediation model for Study 2, in which knowledge about the conflict is controlled for. Non-significant paths are displayed in grey. *p < .05, **p < .01, ***p ≤ .001.
(TIF)
