# Supplementary figures and images for: Engaging in extreme activism in support of others’ political struggles: The role of politically motivated fusion with out-groups
Source: PLoS One. 2018 Jan 5;13(1):e0190639. doi: 10.1371/journal.pone.0190639 (PMC5755793; doi:10.1371/journal.pone.0190639)

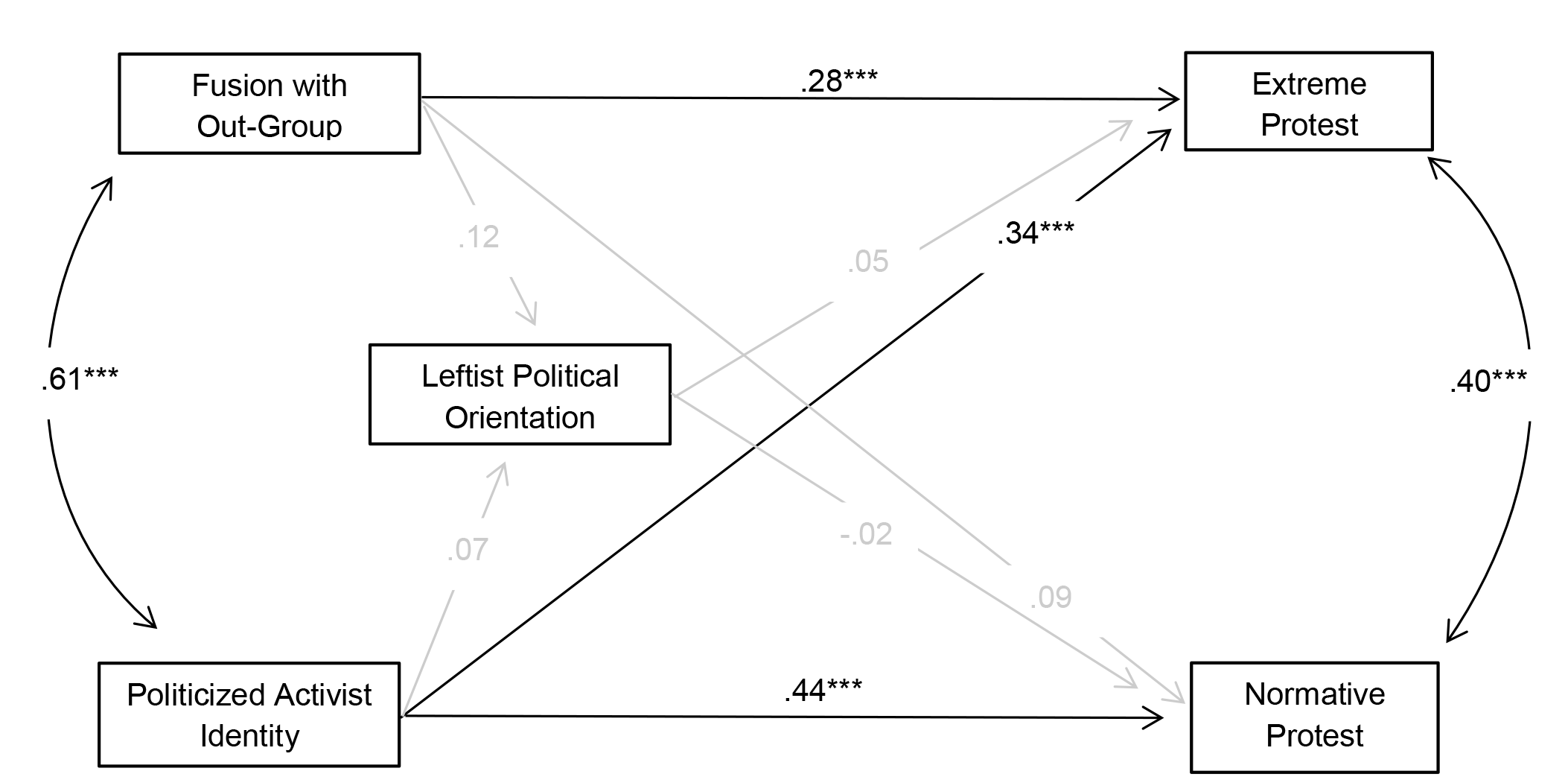

Supplement: S1 Fig — Non-significant paths are displayed in grey. *p < .05, **p < .01, ***p < .001. (TIF) [file pone.0190639.s001.tif]

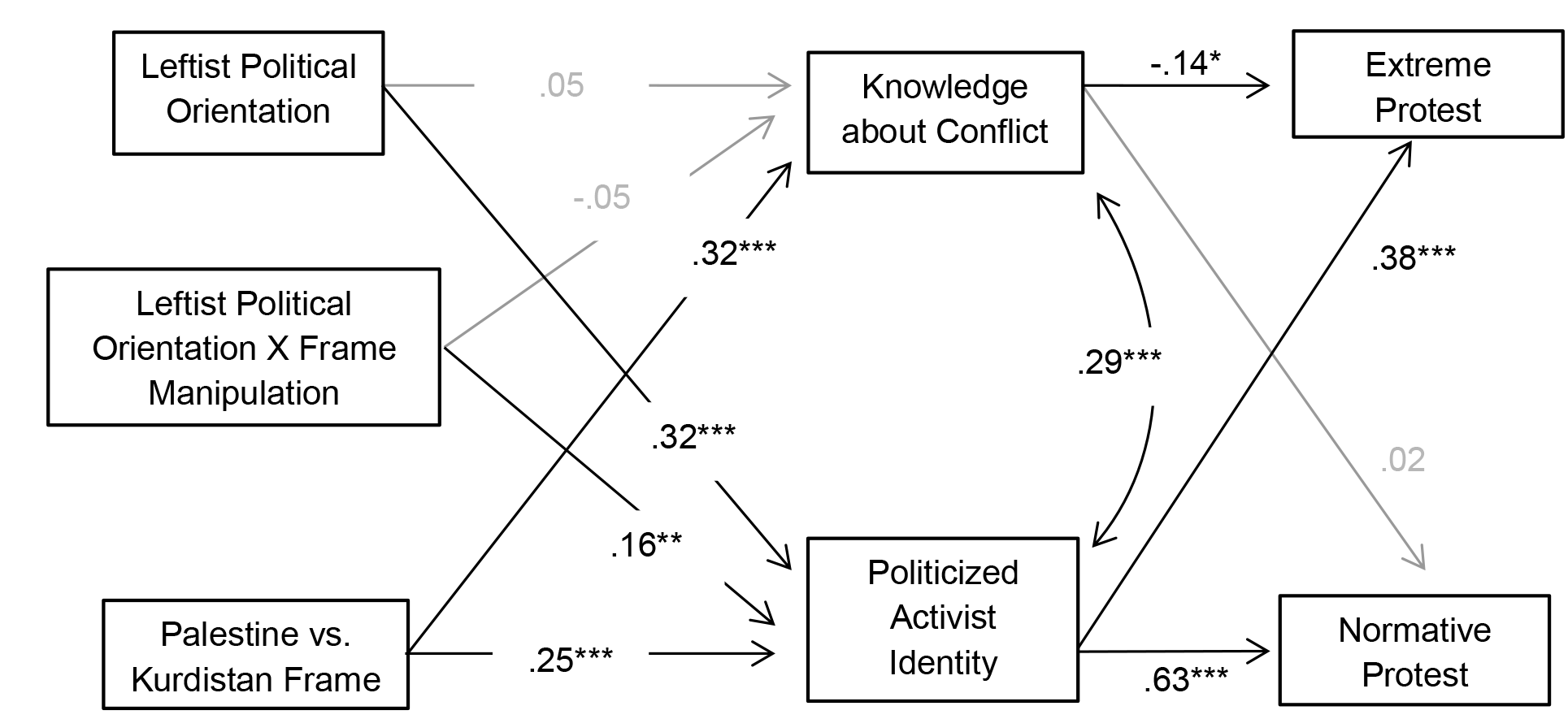

Supplement: S2 Fig — Non-significant paths are displayed in grey. *p < .05, **p < .01, ***p < .001. (TIF) [file pone.0190639.s002.tif]

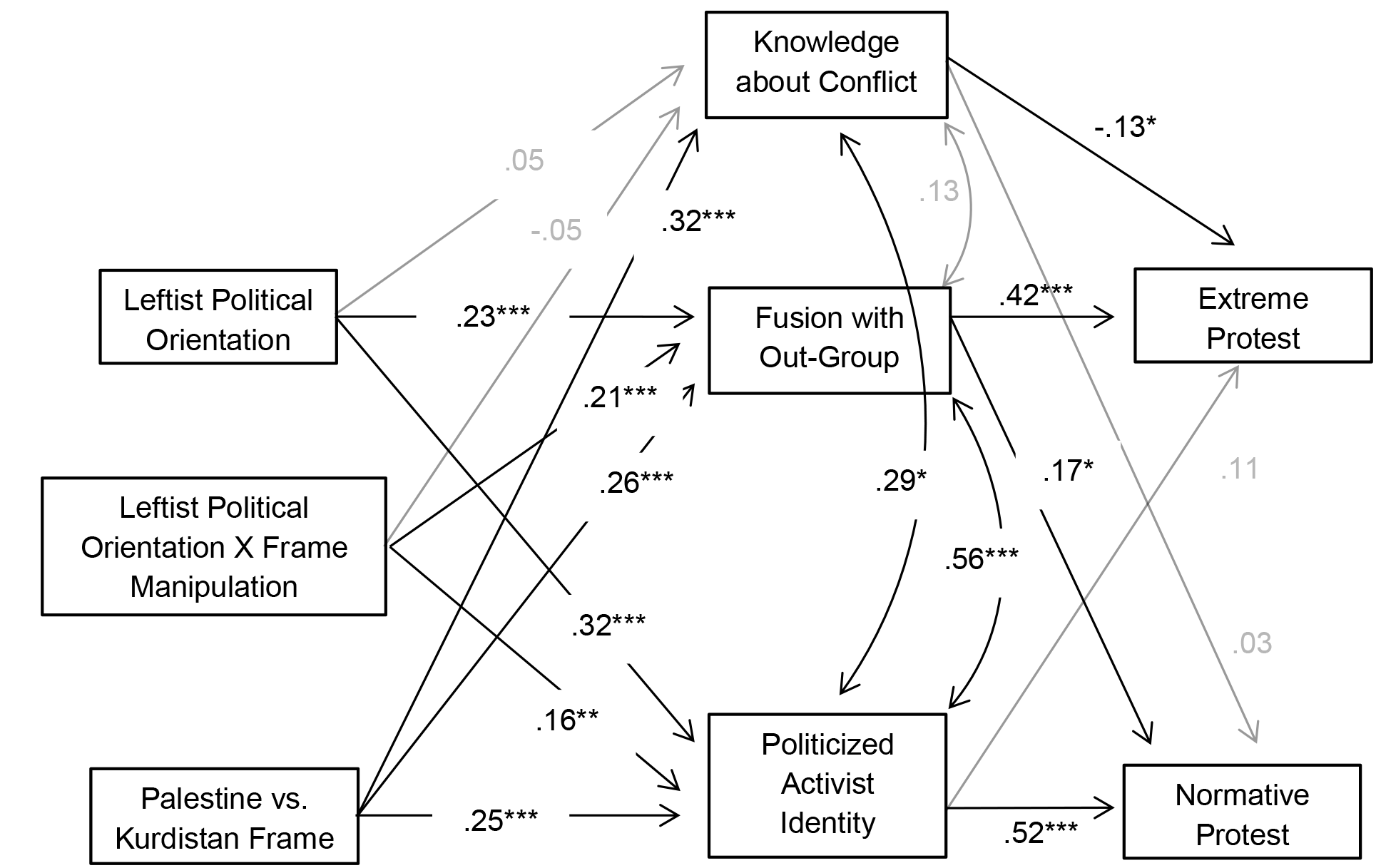

Supplement: S3 Fig — Non-significant paths are displayed in grey. *p < .05, **p < .01, ***p ≤ .001. (TIF) [file pone.0190639.s003.tif]
